# Correction: Discovery of novel METTL3 inhibitors: design, synthesis, and *in silico* and *in vitro* evaluations

**DOI:** 10.1039/d6ra90088g

**Published:** 2026-07-30

**Authors:** Yun Zhang, Mengyan Chen, Yanwen He, Qinlan Chen, Hongdi Han, Xianling Zhang, Yihan Qian, Haiying Jiang, Xiangwei Xu, Zunyuan Wang

**Affiliations:** a Affiliated Yongkang First People's Hospital and School of Pharmacy, Hangzhou Medical College Hangzhou 310013 China wzuny@hmc.edu.cn xuxiangwei@hmc.edu.cn; b School of Public Health, Hangzhou Medical College Hangzhou 310013 China; c School of Clinical Medicine, Hangzhou Medical College Hangzhou 310013 China; d School of Rehabilitation, Hangzhou Medical College Hangzhou 310013 China; e Yongkang First People's Hospital Yongkang 321306 China

## Abstract

Correction for ‘Discovery of novel METTL3 inhibitors: design, synthesis, and *in silico* and *in vitro* evaluations’ by Yun Zhang *et al.*, *RSC Adv.*, 2026, https://doi.org/10.1039/d6ra02851a.

The authors regret that the grant code provided to acknowledge funding from the College Students' Innovation Training Program of Zhejiang Province was incorrect. The correct grant details to be acknowledged are as follows:

This article was funded by the College Students' Innovation Training Program of Zhejiang Province [S202613023014], the Scientific and Technological Innovation (Xinmiao Talents) Program of Zhejiang Province [2026R426A003], Bureau provincial co-construction key project of Zhejiang Administration of Traditional Chinese Medicine [GZY-ZJ-KJ-23060] and Jinhua City Science and Technology Project [2023-4-286].

The Royal Society of Chemistry apologises for these errors and any consequent inconvenience to authors and readers.

